# *In silico* Proteomic Analysis Provides Insights Into Phylogenomics and Plant Biomass Deconstruction Potentials of the Tremelalles

**DOI:** 10.3389/fbioe.2020.00226

**Published:** 2020-04-03

**Authors:** Habibu Aliyu, Olga Gorte, Xinhai Zhou, Anke Neumann, Katrin Ochsenreither

**Affiliations:** ^1^Institute of Process Engineering in Life Science 2: Technical Biology, Karlsruhe Institute of Technology, Karlsruhe, Germany; ^2^State Key Laboratory of Materials-Oriented Chemical Engineering, College of Biotechnology and Pharmaceutical Engineering, Nanjing Tech University, Nanjing, China

**Keywords:** biotechnology, CAZYmes, peptidases, plant biomass degradation, phylogenomics, Tremellales

## Abstract

Basidiomycetes populate a wide range of ecological niches but unlike ascomycetes, their capabilities to decay plant polymers and their potential for biotechnological approaches receive less attention. Particularly, identification and isolation of CAZymes is of biotechnological relevance and has the potential to improve the cache of currently available commercial enzyme cocktails toward enhanced plant biomass utilization. The order Tremellales comprises phylogenetically diverse fungi living as human pathogens, mycoparasites, saprophytes or associated with insects. Here, we have employed comparative genomics approaches to highlight the phylogenomic relationships among thirty-five Tremellales and to identify putative enzymes of biotechnological interest encoded on their genomes. Evaluation of the predicted proteomes of the thirty-five Tremellales revealed 6,918 putative carbohydrate-active enzymes (CAZYmes) and 7,066 peptidases. Two soil isolates, *Saitozyma podzolica* DSM 27192 and *Cryptococcus* sp. JCM 24511, show higher numbers harboring an average of 317 compared to a range of 267–121 CAZYmes for the rest of the strains. Similarly, the proteomes of the two soil isolates along with two plant associated strains contain higher number of peptidases sharing an average of 234 peptidases compared to a range of 226–167 for the rest of the strains. Despite these huge differences and the apparent enrichment of these enzymes among the soil isolates, the data revealed a diversity of the various enzyme families that does not reflect specific habitat type. Growth experiment on various carbohydrates to validate the predictions provides support for this view. Overall, the data indicates that the Tremellales could serve as a rich source of both CAZYmes and peptidases with wide range of potential biotechnological relevance.

## Introduction

Plant biomass is the most abundant carbon rich waste material and can be used by biorefineries for production of food, feed, building block chemicals and bioenergy, such as biofuels. The polymeric plant cell wall comprises cellulose, hemicellulose and pectin as main components while wooden plant material is cross-linked with the aromatic hetero polymer lignin during lignification (Harris and Stone, 2009; Kai et al., 2018). The degradation into monomeric compounds is difficult because of the branched and complex structures (Harris and Stone, 2009; Kai et al., 2018). For instance, pre-treatment is required to deconstruct the complex association of the constituent polymers of the plant biomass prior to hydrolysis into monomers (Harris and Stone, 2009; Rosnow et al., 2017). However, pre-treatment increases processing costs and leads to lower competitiveness with standard fossil fuel (Sheridan, 2013). This process can be augmented biologically by application of various polysaccharide degrading and modifying enzymes, called carbohydrate-active enzymes (CAZymes) (Lombard et al., 2014; Silveira et al., 2015). Thus, finding and isolation of CAZymes is of biotechnological relevance and has the potential to improve the cache of currently available commercial enzyme cocktails toward enhanced plant biomass utilization.

Aside polysaccharides and lignin, plant biomass also consist of substantial amounts (∼5% of the total biomass) of proteins, triglycerides and terpenes (De Schouwer et al., 2019). Considering the estimated global annual biomass production of 146 billion metric tons (Balat and Ayar, 2005), the amount of these secondary fractions is substantial and could be integrated into the overall scheme of biorefining for the production of important industrial chemicals (Bilal et al., 2017; de Paula et al., 2019). Deconstruction of proteins by proteolytic enzymes mainly yields shorter peptides and amino acids that can be utilized in various industries, including food, animal feed, cosmetics, pharmaceuticals, and agrochemicals (De Schouwer et al., 2019. Proteolytic enzymes are among the top industrial enzymes and have been previously reported to account for ∼60% of global enzyme demand (Rao et al., 1998), a market that has been projected to reach a value of $6.32 billion by 2021 (Chapman et al., 2018).

Basidiomycetes populate a wide range of ecological niches, including forest, crops, compost, and plant matter in soils, and are adapted to various substrates. In contrast to ascomycetes, basidiomycetes are less studied for their capabilities to decay plant polymers and their potential for industrial use. The apparent lack of interest in the Basidiomycetes has been linked to the long standing popularity and industrial relevance of the ascomycetes which is well-established rather than the lack of industrially relevant enzymes (Rytioja et al., 2014). Promising candidates for identification of new CAZymes and proteolytic enzymes are fungi isolated from such different biotopes. Recently, a new oleaginous yeasts *Saitozyma podzolica* DSM 27192 has been isolated from peat bog soil (Schulze et al., 2014). Initial analysis of the predicted proteome relative to other members of the order Tremellales revealed that DSM 27192 genome encodes a larger number of proteins linked to carbohydrate-active and proteolytic enzymes (Aliyu et al., 2019).

Previous genomic studies on the distribution of plant biomass hydrolyzing enzymes among fungi (Zhao et al., 2014) have largely paid less attention to the Tremellales. Similarly, the contribution and identification of putative enzymes for the hydrolysis of the protein component of the plant biomass remained understudied (De Schouwer et al., 2019) compared to polysaccharides. Here, we have employed phylogenomic and comparative genomic strategies to study thirty-five members of the order Tremellales. We used genome scale phylogenetic analysis to decipher the genetic diversity among members of the order. In-depth comparison of the predicted proteomes with emphasis on sources of isolation identified a diverse repertoire of CAZymes and peptidases and revealed an enrichment of these features among the two-soil isolated *Saitozyma* species. This study, therefore, enhances the understanding of both the evolutionary and functional diversities of the Tremellales, which will be useful for the development of strategies for future biotechnological and industrial exploration of these fungi.

## Materials and Methods

### Genome Sequences, Structural and Functional Annotations

The genomes of thirty-five members of the order Tremellales and one out group strain from the order Trichosporonales (Table 1), obtained from the NCBI or JGI databases, were structurally annotated using the Funannotate pipeline (v. 1.5.0-8f86f8c) (Love et al., 2018). The completeness of each genome was determined using BUSCO v3.0.3 (Waterhouse et al., 2018). To identify putative carbohydrate-active enzymes (CAZYmes), the proteomes of the strains were annotated using run_dbcan 2.0, a dbCAN standalone algorithm comprising three tools; DIAMOND, Hotpep, and HMMER (Zhang et al., 2018). To improve CAZYmes prediction, this study focuses only on sequences identified by at least two of these tools (Zhang et al., 2018). Proteolytic enzymes were identified using BLASTP search (*E*-value cut-off ≤1.00E-10) with the proteomes of the strains as queries against MEROPS database release 12.1 (Rawlings et al., 2017). Major protein families of sugar and amino acid transporters were identified from Interproscan v.5.30-69.0 (Jones et al., 2014) annotation of the various proteomes.

**TABLE 1:** Genomic features of thirty-five Tremellales and one outgroup strain included in this study.

**Strains**	**Isolation source/locality**	**Assembly name**	**Assembly Size**	**# Scaffolds**	**Scaffold N50**	**% GC**	**# Genes**	**# Proteins**	**# tRNA**	Com*	**Dup^¥^**
***Cryptococcus gattii* WM276**	*Eucalyptus tereticornis* debris, Australia	ASM18594v1	18,374,760	14	1,333,124	47.85	6,570	6,429	141	97.6	0.0
***Cryptococcus neoformans* var. *grubii* H99**	Human	CNA3	18,916,112	15	1,422,463	48.21	6,849	6,694	155	96.2	0.7
***Cryptococcus neoformans var. neoformans* JEC21**	Generated from NIH B-3501 and NIH B-3502 α-types (progenies of ATCC 28957 × ATCC 28958) (https://www.ncbi.nlm.nih.gov/pmc/articles/-PMC257671/pdf/iai00026-0294.pdf)	ASM9104v1	19,051,922	14	1,438,950	48.54	6,879	6,740	139	96.9	0.7
***Cryptococcus floricola* DSM 27421**	Flower nectar collected in Tenerife, Canary Islands, Spain (https://mbio.asm.org/content/10/3/e00764-19)	ASM635230v1	21,743,217	16	1,629,715	53.47	7,703	7,511	192	93.1	0.7
***Cryptococcus wingfieldii* CBS 7118^*T*^**	Frass from scolytid beetles, South Africa	ASM614915v1	20,871,786	15	1,575,190	53.40	7,412	7,217	195	94.8	1.0
***Cryptococcus amylolentus* CBS 6039^*T*^**	Frass of larvae of *Enneadesmus forficulus* (buprestid beetle), South Africa	Cryp_amyl_CBS6039_V3	20,254,996	15	1,545,655	53.36	8,357	8,164	193	96.2	1.0
***Cryptococcus amylolentus* CBS 6273**	Frass of *Sinoxylon ruficorne* in *Dichrostachys cinerea*, South Africa	Cryp_amyl_CBS6273_V2	20,280,838	18	1,529,651	53.17	8,350	8,175	175	93.1	0.3
***Cryptococcus depauperatus* CBS 7841^*T*^**	Dead spider, Canada	Filo_depa_CBS7841_V1	15,797,590	68	501,291	44.36	6,251	6,125	126	96.9	0.3
***Cryptococcus depauperatus* CBS 7855**	Surface of colony of *Verticillium lecanii*, from dead caterpillar of *Carpocapsa pomonella*, Czechia	Filo_depa_CBS7855_V2	15,656,276	41	882,956	44.73	6,232	6,106	126	92.4	0.7
***Kwoniella heveanensis* BCC8398**	Insect frass, Thailand (CBS 12232)	Cryp_heve_BCC8398_V1	25,469,355	67	709,378	51.85	8,039	7,957	82	91.7	0.3
***Kwoniella heveanensis* CBS 569^*T*^**	Sheet rubber, Indonesia	Cryp_heve_CBS569_V2	25,253,281	242	174,698	51.78	7,939	7,853	86	92.1	0.7
***Kwoniella dejecticola* CBS 10117^*T*^**	Frass of the litchi fruit borer *Conopomorpha sinensis*, Vietnam	Cryp_deje_CBS10117_V1	23,862,392	13	2,116,304	48.38	8,519	8,445	74	94.1	0.3
***Kwoniella pini* CBS 10737^*T*^**	Dead needles of *Pinus sylvestris* in a mixed forest, Russia	Cryp_pinu_CBS10737_V1	20,828,584	18	1,626,097	40.20	7,714	7,639	75	94.8	0.7
***Kwoniella bestiolae* CBS 10118^*T*^**	Frass of the litchi fruit borer *Conopomorpha sinensis*, Vietnam	Cryp_best_CBS10118_V1	24,360,772	12	3,422,270	47.22	9,068	8,992	76	96.2	0.3
***Kwoniella mangroviensis* CBS 10435**	Seawater, Bahamas	Kwon_mang_CBS10435_V2	22,653,759	37	1,966,685	44.78	8,324	8,243	81	94.1	0.7
***Kwoniella mangroviensis* CBS 8507^*T*^**	Seawater	Kwon_mang_CBS8507_V2	22,654,511	62	1,048,156	44.76	8,326	8,249	77	95.5	0.0
***Kwoniella mangroviensis* CBS 8886**	Seawater (mangrove), Bahamas	Kwon_mang_CBS8886_V1	22,872,713	41	2,035,775	44.53	8,342	8,264	78	95.2	0.0
***Saitozyma podzolica* DSM 27192**	Peat bog soil, Germany	ASM394221v1	29,888,215	46	1,066,819	58.79	10,312	10,224	88	91.4	0.0
***Cryptococcus* sp. JCM 24511**	Soil, Japan	JCM_24511_assembly_v001	28,180,937	55	1,281,587	58.53	9,890	9,835	55	94.8	0.0
***Dioszegia crocea* JCM 2961^*T*^**	Strawberries, United Kingdom	JCM_2961_assembly_v001	20,595,339	26	1,954,963	53.18	8,685	8,639	46	94.8	1.0
***Dioszegia aurantiaca* JCM 2956^*T*^**	Over-wintered nettle stems of *Urtica* sp., Canada	JCM_2956_assembly_v001	19,344,119	52	1,282,637	53.56	7,883	7,835	48	93.1	0.7
***Tremella mesenterica* ATCC 28783**	*Alnus rubra*, Canada	Trem_mese_ATCC28783_V1	27,110,854	295	180,988	41.28	7,348	7,309	39	93.8	1.0
***Tremella mesenterica* DSM 1558**	*Alnus rubra*	Treme1	28,639,919	45	1,622,698	45.67	8,548	8,513	35	87.6	0.7
***Tremella fuciformis* tr26**	(derived from *T. fuciformis* T0040) *Annulohypoxylon stygium*, China	ASM98790v1	23,635,607	3,502	18,448	57.03	10,769	10,713	56	88.6	0.7
***Fibulobasidium inconspicuum* Phaff 89-39**	Unknown (https://phaffcollection.ucdavis.edu/search-online-ordering)	Phaff89-39v1.0	20,291,509	31	1,209,751	59.08	7,944	7,900	44	92.4	1.0
***Naematelia encephala* UCDFST 68-887.2**	Isolated from cracked bark of *Salix* sp., Canada (https://phaffcollection.ucdavis.edu/search-online-ordering)	Treen1	19,786,307	151	209,500	49.30	7,674	7,631	43	87.3	0.7
***Fellomyces penicillatus* Phaff 54-35**	Isolated from Hawiian pineapple (https://phaffcollection.ucdavis.edu/search-online-ordering)	Phaff54-35v1.0	21,049,006	22	2,827,852	47.59	8,094	8,055	39	96.6	0.7
***Kockovaella imperatae* NRRL Y 17943**	Leaf of cogon, *Imperata cylindrica* Thailand (https://nrrl.ncaur.usda.gov/cgi-bin/usda/yeast/report.html?nrrlcodes=Y%2d17943)	Kocim1	17,465,713	38	1,071,374	52.20	7,122	7,091	31	87.2	0.3
***Bullera alba* JCM 2954^*T*^**	Air in a dairy, United States	JCM_2954_assembly_v001	19,417,308	8	2,864,929	54.31	7,835	7,794	41	96.9	0.3
***Sirobasidium intermedium* CBS 7805**	*Eutypella leprosa* on twig of *Ulmus* sp. (elm tree), United Kingdom	CBS7805v1.0	21,959,807	102	832,995	56.14	9,016	8,966	50	92.0	1.0
***Papiliotrema flavescens* NRRL Y 50378**	Derived from OH182.9 (https://www.ars.usda.gov/research/publications/publication/?seqNo115=237942) Wheat anther, United States (https://www.sciencedirect.com/science/article/pii/S2352407316300348#bb0055)	Cf_30_300r_Split10plusN	22,790,521	712	71,416	58.46	9,328	9,262	66	91.4	0.7
***Papiliotrema laurentii* RY1**	Food (Kombucha tea); India	ASM73882v1	19,145,913	1,152	32,353	56.13	7,687	7,584	103	91.3	0.3
***Phaeotremella skinneri* JCM 9039^*T*^**	Insect frass beneath the bark of hemlock *Tsuga heterophylla*, United States	JCM_9039_assembly_v001	20,788,256	58	1,003,985	50.23	7,471	7,423	48	95.9	0.7
***Phaeotremella fagi* JCM 13614**	Rotten beech, Netherlands	JCM_13614_assembly_v001	22,649,946	30	1,602,030	42.43	10,146	10,089	57	94.5	0.7
***Cryptococcus* sp. 05/00**	Rainbow hydrothermal site on the Mid-Atlantic Ridge	Mo29	23,801,231	687	165,604	53.09	8,179	7,984	195	91.4	0.7
***Cutaneotrichosporon oleaginosum* ATCC 20509^*T*^**	Dairy plant	ASM171244v1	19,908,169	16	250,97,47	60.59	8,302	8,036	266	92.0	0.3

*Completeness and ^¥^duplication determined using BUSCO based on fungi_odb9.

### Phylogenomic Analysis

Orthologous relationships among the predicted protein sequences of the thirty-five Tremellales and the outgroup strains were inferred using OrthoFinder v2.3.3 (Emms and Kelly, 2015) with default parameter settings. To construct the phylogeny of Tremellales, single copy orthologs, were aligned using T-coffee v11.00.8cbe486 (Notredame et al., 2000; Magis et al., 2014). The resultant alignment was concatenated and trimmed using Gblocks v0.9b (Castresana, 2000; Talavera and Castresana, 2007). The trimmed alignment was used to construct a Maximum likelihood (ML) tree using IQ-TREE version 1.6.7 (Schmidt et al., 2014) based on the LG + F + R6 model (predicted using IQ-TREE) and 1,000 bootstrap replicates. Average amino acid identity (AAI) and orthologous average nucleotide identity (orthoANI) among the compared strains were computed using CompareM^1^ and OrthoANIu tool (Yoon et al., 2017).

### Carbon Substrate Utilization

*Saitozyma podzolica* DSM 27192 was recently deposited at the DSMZ culture collection, Germany (Schulze et al., 2014). *Tremella mesenterica* DSM 1558 was obtained from DSMZ. *Cutaneotrichosporon oleaginosum* ATCC 20509 was acquired from ATCC Culture Collection, United States. *Dioszegia aurantiaca* CBS 6980, *Kwoniella mangrovensis* CBS 8507, *Sirobasidium intermedium* CBS 7805 and *Cryptococcus amylolentus* CBS 6039 were purchased from CBS, Netherlands. *Cryptococcus* sp. JCM 24511 and *Cryptococcus fagi* JCM 13614 were ordered from JCM, Japan.

The ability of isolates to metabolize certain carbohydrates was characterized with a standardized API 50 CHL system (BioMérieux, Nürtingen, Germany) consisting of 50 biochemical tests. The strains were activated in liquid culture containing YM medium (3 g/L yeast extract, 3 g/L malt extract, 5 g/L peptone, pH 7, sterile supplemented with 10 g/L glucose after autoclaving). After 24 h of growth OD_600 nm_ was determined. The cultures were washed twice with sterile saline (0.9% w/v, NaCl) and the pellets were resuspended in sterile distilled water containing 0.17 g/L bromocresol purple to an OD_600 nm_ = 1. Each isolate suspension was applied into the pockets of the API 50 CH. Strips were moistened and covered as recommended by the manufacturer and incubated at optimal growth temperature of the respective strain (20, 25, or 30°C). Colorimetrical changes were recorded after the first 3 days and verified after 7 and 10 days.

For the plate-based tests, YNB medium (HP26.1; Carl Roth) was prepared according to manufacturer’s protocol with 15 g/L agar. Xylan from beechwood (4414.4 Carl Roth), xylan from corncobs (8659.3 Carl Roth), inulin (I2255; Sigma-Aldrich), cellulose (Avicel PH-101; 11365; Sigma-Aldrich), starch (S4126; Sigma-Aldrich), carboxymethylcellulose (CMC; C5013; Sigma-Aldrich), pectin (93854; Sigma-Aldrich), chitin (8845.1; Sigma-Aldrich), N-acetyl-D-glucosamine (8993.2; Carl Roth), D(+)-Glucosamine (3769.1 Carl Roth) and D-Glucose monohydrate as positive control, were dissolved in distilled water, autoclaved separately and supplemented to the medium to a final concentration of 10 g/L. As negative control agar without carbon source was prepared. All nine strains were activated in YM medium, as described above, and washed twice with sterile 0.9% NaCl. The pellets were then subsequently resuspended in saline to an OD_600 nm_ = 1. Each agar plate was inoculated with three 10 μL of strain suspension. The plates were incubated at the optimal growth temperature (20, 25, and 30°C) and monitored for 10 days.

Principal component analysis (PCA) plots and heatmaps are generated using Clustvis web tool (Metsalu and Vilo, 2015) while correlation analyses and visualization were conducted in R 3.5.2 using packages corrplot (Wei et al., 2017) and PerformanceAnalytics (Peterson et al., 2018).

## Results

### Genome Characteristics and Niche Specialization Among the Tremelalles

A survey of the NCBI database showed that as at October, 2019, 106 genome assemblies of strains affiliated with Tremellales are public. Majority of these assemblies are from the family *Cryptococcaceae* (93; ∼ 88%), with isolates of *Cryptococcus neoformans and C. gattii* comprising 54 (∼51%) and 20 (∼19%), respectively, of the total available sequences. The present study, however, focuses on genome sequences of thirty-five isolates from eleven families of Tremellales, including one genome sequence each of *C. gattii* WM276, *C. neoformans* var. *grubii* H99 and *C. neoformans* var. *neoformans* JEC21 selected as proxies for the various species complexes to which they belong. The genome of *Cutaneotrichosporon oleaginosum* ATCC 20509^*T*^ (order Trichosporonales) was included as an outgroup (Table 1). The sizes of the thirty-five studied genomes range between 15.66 Mb (*C. depauperatus* CBS 7855) and 29.88 Mb (*Saitozyma podzolica* DSM 27192) base pairs and code between 6,232 (*C. depauperatus* CBS 7855) and 10,769 (*Tremella fuciformis* tr26) genes including between 31 (*Kockovaella imperatae* NRRL Y 17943) and 195 (*C. wingfieldii* CBS 7118^*T*^) tRNA genes. These genome sizes fall within the lower end of reported fungal genomes (8.97–177.57 Mb) and well below the average of those of Basidiomycota which has been estimated at 46.48 Mb (Mohanta and Bae, 2015). The number of protein coding genes ranged between 6,106 in *Cryptococcus depauperatus* CBS 7855 and 10,713 in *Tremella fuciformis* tr26 both which are lower than the average of ∼15,432 protein models reported for Basidiomycota. However, tr26 genome shows the highest contigs number of 3,502, which may be indicative of a highly fragmented genome sequence. Strain tr26 is also characterized by low level of genome completeness (∼88.6%). However, based on BUSCO (Waterhouse et al., 2018) fungi_odb9 single copy orthologs, genome completeness among the studied strains varied between 87.2% in *Kockovaella imperatae* NRRL Y 17943 (38 contigs) and 97.6% in *C. gattii* WM276 (14 contigs). The latter isolate along with *S. podzolica* DSM 27192, *Cryptococcus* sp. JCM 24511, *Kwoniella mangroviensis* CBS 8886 and *K. mangroviensis* CBS 8507 showed zero evidence of genome duplication while the rest of the studied strains contain low level of duplications, ranging between 0.3 and 1%. Its noteworthy, however, that this work reports only the genome properties of the monokaryotic (asexual) yeast state of these fungi.

With reference to their sources of origin (Table 1), the studied isolates show wide distribution with strains isolated from dairy (1), fungi (3), human (2), insect frass (7), plant (11), soil (2), sea (4) and one isolate each from dead spider, sheet rubber, rotten beech and kombucha tea. The origin of *Fibulobasidium inconspicuum* Phaff 89-39 is unknown. Thus, considering their preferred habitat and views from various literature (Findley et al., 2009; Millanes et al., 2011; Yurkov et al., 2015; May et al., 2016), the strains have been classified into eight groups, namely mycoparasitic, pathogenic, saprobic (arthropod frass, dead arthropods, plants, and others), sea- and soil -inhabiting.

### Phylogenomics of the Tremelalles

**FIGURE 1:**
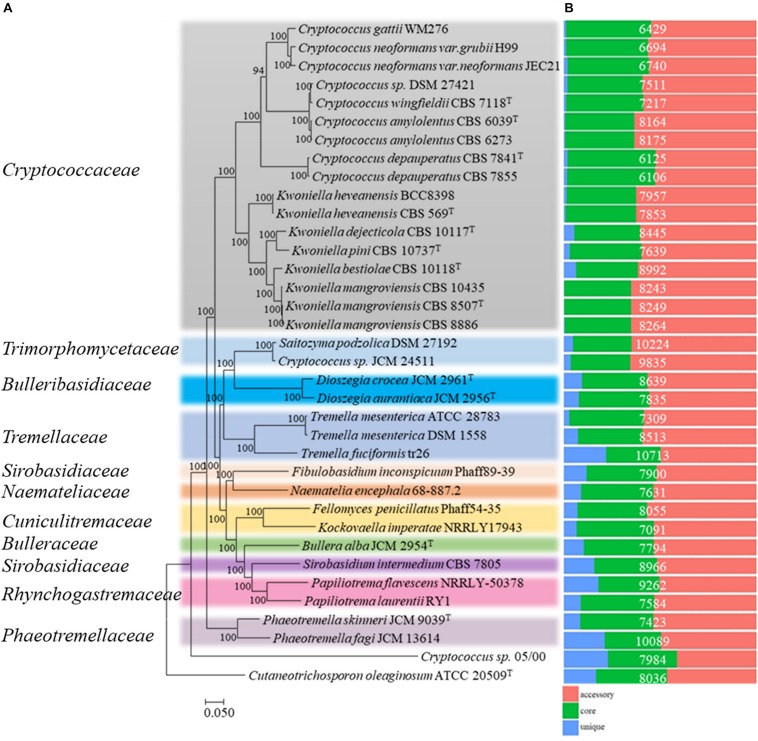
Phylogenomic analysis of thirty-five members of the order Tremellales. **(A)** Maximum likelihood (ML) tree inferred from the concatenated protein alignment (412,000 amino acids) of 1,597 single copy proteins. The phylogeny was generated using IQ-TREE version 1.6.7 based on the LG + F + R6 model. The ML was constructed with confidence values based on 1,000 bootstrap replicates. Various families of the order have been indicated and colored on the tree for clarity. **(B)** Orthologous relationships of the predicted proteins among the studied strains. The proteome size for each strain is indicated in white fonts.

To place the studied strains into phylogenomic perspective, the orthologous relationships among the predicted proteome (289,686 proteins) of the thirty-five Tremelalles and the outgroup strain were determined using OrthoFinder (Emms and Kelly, 2015). Analysis of the protein families (orthogroups) revealed 269,580 (93.1%) proteins are assigned in 13,504 orthogroups of which 122 (614; 0.2% proteins) are strain specific. Evaluation of the orthologous proteome identified 2,634 orthogroups (104, 039; ∼36% proteins) are core to all the studied strains. Of these, 1,597 comprise the single copy orthogroups (SCO), representing between 15 and 25% of the individual proteomes of the studied fungi. To infer phylogenetic relationships among the studied Tremellales, a maximum likelihood (ML) tree (Figure 1A) was generated from a concatenated and trimmed alignment of the SCO proteins comprising 412,000 amino acids using IQ-TREE (Schmidt et al., 2014; Chernomor et al., 2016). Based on this tree, *Cryptococcus* sp. 05/00 clusters with the outgroup strain suggesting a distant relationship with the strains in the order Tremellales. This strain also harbors greater proportion of unique proteins (Figure 1B) relative to the other strain, indicating disparate evolutionary history. Aside this, members of the family *Phaeotremellaceae* (*Phaeotremella* spp.) form a monophyletic clade distinct from the rest of the Tremellales which are placed in two separate major clusters. The first cluster comprises members of the genera *Cryptococcus* and *Kwoniella* while the second comprises ten genera from different families, including two *Saitozyma* spp. The latter cluster includes strains harboring greater number of strains specific proteins (Figure 1B). To highlight the relationships at various taxa levels, average amino acid identity (AAI) values were determined among the thirty-five Tremellales (Supplementary Figure S1A). Excluding *Cryptococcus* sp. 05/00, the AAI between the compared strains ranged between 60.7 (*Kockovaella imperatae* NRRL Y 17943 and *Dioszegia aurantiaca* JCM 2956^*T*^) and 98.72 (*Cryptococcus amylolentus* CBS 6273 and C. *amylolentus* CBS 6039^*T*^). The *Cryptococcus* clade incorporates strains whose AAI ranged between 61.36 and 98.71% while isolates in the *Saitozyma* clade share AAI ranging between 56.15 and 92.67%, further confirming the greater diversity in the latter clade. Within the subclades, the *Cryptococcus* spp. share an AAI range of 64.61–98.71% with C. *gattii* WM276 and *C. neoformans* sharing an AAI between 86.28 and 90.02% and *C. amylolentus*, *C. wingfieldii* and *C. floricola* sharing between 91.62 and 98.71% AAI. Furthermore, the *Kwoniella* spp. show an AAI ranging between 67.28 and 97.98%. On the other hand, majority of the strains in *Saitozyma* clade form monophyletic branches with exception of *Saitozyma*, *Dioszegia*, and *Tremella* spp. for which the strains within each branch share an AAI of 88.18, 77.35, and 92.67%, respectively. To further ascertain the genetic relationship of strains at species level, average nucleotide identity (ANI) values among closely related isolates were computed (Supplementary Figure S1B). The ANI among C. *gattii* WM276 and *C. neoformans* ranged between 83.60 and 88.31% while *C. amylolentus*, *C. wingfieldii* and *C. floricola* shared ANI that range of 93.24–99.59%. In contrast, ANI of 86.52% is shared between *Saitozyma podzolica* DSM 27192 and *Cryptococcus* sp. JCM 24511 both of which have been affiliated with *Saitozyma podzolica*.

### Genome Wide Comparisons of Carbohydrate-Active Enzymes and Proteolytic Enzymes

#### Proteolytic Enzymes

**FIGURE 2:**
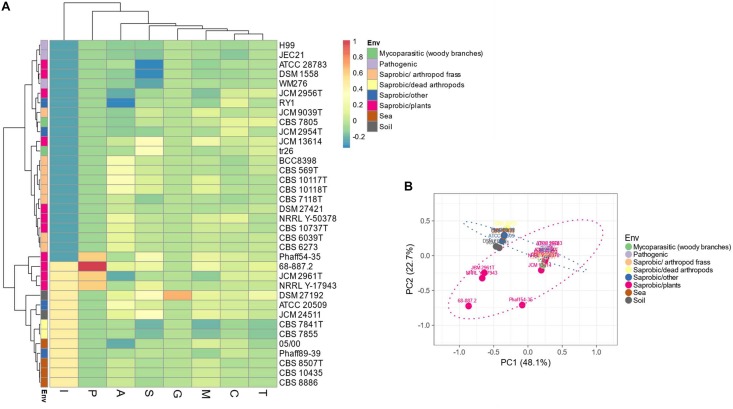
Distribution of peptidases and peptidase inhibitors among the Tremellales species. **(A)** Heat map showing the distribution of the peptidases and peptidase inhibitors. Values are ln(*x* + 1)-transformed and rows and columns clustered using Euclidean distance and Ward linkage. **(B)** Principal components analysis showing relationships among studied strains (including isolation sources) based on the distribution of peptidases and peptidase inhibitors. The ellipses are predicted to indicate probability (0.95) that a new observation from the same group will fall inside the ellipse. A: aspartic peptidases, C: cysteine peptidases, G: glutamic peptidases, M: metallo-peptidases, P: mixed peptidases, S: serine peptidases, T: threonine peptidases, and I: protease inhibitors.

Evaluation of the predicted proteomes of thirty-five Tremellales revealed 7,079 (average: ∼197) peptidases and peptidase inhibitors (Supplementary Table S1). Genomes of *C. depauperatus* CBS 7841^*T*^ isolated from a dead arthropod and *Tremella mesenterica* DSM 1558 isolated from plant code for the least peptidases of ∼85% of the average peptidase number and those of *S. podzolica* DSM 27192 isolated from soil and *Phaeotremella fagi* JCM 13614 isolated from rotten beech harbor the highest number of peptidases; ∼120% of the average. Clustering of the predicted peptidases, including information on isolation sources among the studied fungi revealed two major clusters in which the sea-, soil-dwelling and dead arthropods isolates group distinctly from the arthropod frass isolates while strains of fungal and plant sources are distributed across both clusters. However, the plant associated strains within soil isolates clade show distinct clustering (Figures 2A,B).

**FIGURE 3:**
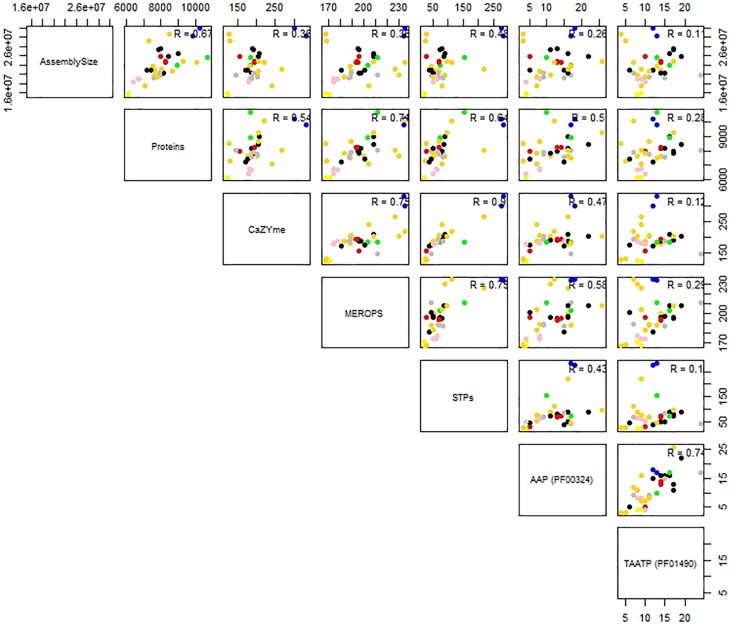
Correlation analysis of genome and proteome sizes, and predicted CAZYmes, peptidases, sugar, and amino acid transporters of thirty-five Tremellales. Coefficient of correlation *R* values indicates the strength of the association and niche specialization of the strains are color coded; black, blue, cyan, gold, green, gray, pink, red representing saprobic (arthropods frass), soil, saprobic (dead arthropods), saprobic (plants), mycoparasitic, saprobic (others), pathogenic, sea isolates, respectively.

Predictions for possible explanation for the observed differences in peptidase abundance among the Tremellales revealed a significant (*P* < 0.01) and strong (*r* = 0.71) correlation between proteome size and protease abundance (Figure 3 and Supplementary Figure S2). Despite the significant (*P* < 0.01) correlation between genome and proteome sizes, the former show no significant association with peptidases number. Similarly, habitat type appears to contribute to peptidase abundance with the soil and certain saprobic plant associated strains clearly harboring higher numbers of peptidases. Further, the proteomes were queried for two families of amino acid transporters, namely amino acid permease (PF00324) and transmembrane amino acid transporter protein (PF01490). Evaluation of these transporters revealed that only the former shows significant (*P* < 0.01) correlation with protease abundance (Supplementary Figure S2). The predicted inhibitors of peptidases (I) and two peptidase families (G and P) are uniquely associated with specific strains in the soil isolates cluster (Figures 2A,B and Supplementary Table S1). A putative aspergilloglutamic peptidase (G01) is uniquely present in *S. podzolica* DSM 27192 while the mixed peptidase, P01 (putative β-aminopeptidases), is exclusive found among the plant associated isolates of the group; *Naematelia encephala* UCDFST 68-887.2, *Dioszegia crocea* JCM 2961^*T*^, *K. imperatae* NRRL Y 17943 and *Fellomyces penicillatus* Phaff 54-35. The genome of UCDFST 68-887.2 encodes homologs of both BapF peptidase and DmpA aminopeptidase, JCM 2961^*T*^ and Phaff54-35 genomes encode only putative BapF peptidase and NRRL Y 17943 genome harbors only DmpA aminopeptidase. Both enzymes have been suggested to have similar substrate specificity (John-White et al., 2017). The predicted peptidase inhibitors include I32 (survivin) present in the two soil isolates (*S. podzolica* DSM 27192 and *Cryptococcus* sp. JCM 24511), the hydrothermal isolate (05/00) and Phaff89-39 which is of unknown origin while I51 (serine carboxypeptidase Y inhibitor) was identified in eight strains including three each isolated from plants and sea, and one each from a dead spider and fungi.

##### Aspartic peptidases (APs)

Evaluation of the peptidase sets (Supplementary Table S1) predicted from the proteomes of the studied isolates revealed only three putative APs belonging to the families A01A (pepsin A), A22B (impas 1 peptidase) and A28A (DNA-damage inducible protein 1). The latter two families occur in single copies in all studied strains while the number of A01A ranged between 6 and 14. Although, the distribution of A1 differs among isolates of similar habitat types, this peptidase is more prevalent among isolates associated with arthropods frass relative to the pathogenic strains (Figures 2A,B and Supplementary Table S1).

##### Cysteine peptidases (CPs)

Twenty putative CPs families have been identified in the proteomes of the studied isolates with members of four families, namely C13 (putative glycosylphosphatidylinositol: protein transamidase), C54 (autophagin-1), C85 (OTUD5 peptidase), C86 (ataxin-3) being shared by all strains (Figures 2A,B and Supplementary Table S1). Further three families, C50 (separase), C65 (otubain), and C78B (UfSP1 peptidase) are common to most of the strains while orthologs of C45 (acyl-coenzyme A:6-aminopenicillanic acid acyl-transferase precursor) and C110 (kyphoscoliosis peptidase) are rare, occurring in one strain each. On the other hand, C19 (ubiquitin-specific peptidase 14) and C26 (gamma-glutamyl hydrolase) are the most abundant C-peptidases represented at an average of 14 and 9 proteins, respectively, in each strain. While there appears to be no pattern of association of specific C-peptidase families with fungal lifestyles (Supplementary Figure S3A), certain plant associated saprobes contain relatively higher numbers of C26 compared to the pathogenic strains (Supplementary Figure S3A and Supplementary Table S1). Conspicuously, three pathogenic, two soil, four arthropods frass (CBS 10117^*T*^, CBS 6039^*T*^, CBS 6273, and CBS 7118^*T*^), two dead arthropods and two plant (CBS10737^*T*^, DSM27421) associated isolates lack homologs of C01B (bleomycin hydrolase). Similarly, C115 (MINDY-1 protein) was restricted to 05/00, JCM 9039T and JCM 13614 while, DSM 27192, JCM 24511 (soil), JCM 2961T, 68-887.2 and Phaff54-35 harbor homologs of C15 (pyroglutamyl-peptidase I). Evaluation of the association between C-peptidases and genome features revealed no association with genome size (Supplementary Figures S4A,B). However, there was significant but week correlation between number C-peptidases and proteome sizes with the smaller proteomes harboring fewer C-peptidases.

##### Metallopeptidases (MPs)

The MPs represent the most abundant and diverse proteases harboured by the studied Tremellales (Figures 2A,B and Supplementary Table S1). Analysis of the predicted proteases identified twenty-three distinct families of M-peptidases including M13 (neprilysin) and M14A (carboxypeptidase A1) occurring in single copies and M41 (FtsH peptidase) in duplicate copies in all the studied strains. On the average, 64 (range: 52–78) M-peptidases are encoded on the genomes of the studied isolates with M20 (putative glutamate carboxypeptidase) and M24 (methionyl aminopeptidase) being the most prevalent families with an average of 9 (range: 3–17) and 9 (range: 9–11) proteins, respectively. The distribution of M19 (membrane dipeptidase), M20 and M38 (isoaspartyl dipeptidase) seems to distinguish the soil, majority of the sea and arthropods frass from the pathogenic and mycoparasitic isolates (Supplementary Figure S3b). Strains JCM 2956T and Phaff89-39 uniquely harbor homologs of M06 (myroilysin) and M10B (membrane-type matrix metallopeptidase-6), respectively. Interestingly, only proteomes of three plant isolates, 68-887.2, NRRL Y 17943 and Phaff54-35 contain the putative M81 (microcystinase MlrC) while 05/00 harbors large numbers of M03A (thimet oligopeptidase).

##### Serine peptidases (SPs)

Prediction of the putative peptidases revealed 12 families of serine peptidases are encoded in the various genomes of Tremellales at an average of 55 (range: 38–77) proteins per genome. Members of the families S09 (prolyl oligopeptidase) and S33 (prolyl aminopeptidase) are the most prevalent S-proteases with an average of 20 (range: 12–35) and 14 (range: 10–24) proteins, respectively, while S14 (peptidase Clp), S28 (lysosomal Pro-Xaa carboxypeptidase), and S59 (nucleoporin 145) show similar distribution among all the studied strains (Supplementary Table S1).

##### Threonine peptidases (TPs)

Of the 6 known T-peptidases, three have been identified in the genomes of the studied Tremellales with an overall average of 19 (range: 16–23, Supplementary Table S1) proteins. T05 (ornithine acetyltransferase precursor) occur in one copy in all but one strain while T01 (proteasome, beta component) family is overrepresented with 14 putative orthologs in all strains except DSM 27192 which contain 16 T01 proteins (Supplementary Figure S3D).

#### Carbohydrate-Active Enzymes

**FIGURE 4:**
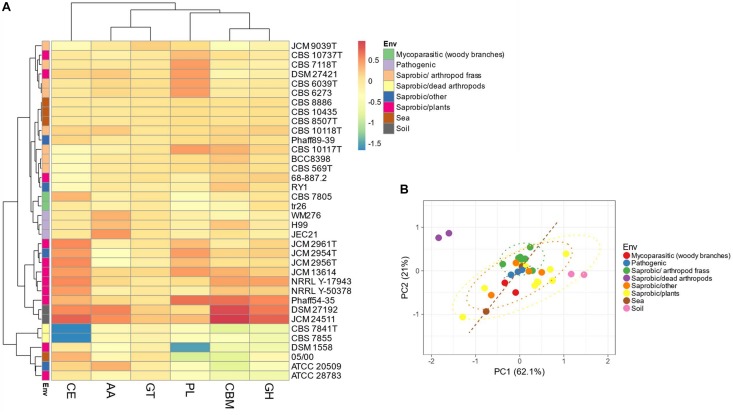
Distribution of predicted CAZYmes among the Tremellales species. AA: auxiliary activities, CBM: carbohydrate-binding modules, CE: carbohydrate esterases, GH: glycoside hydrolases, GT: glycosyltransferases and PL: polysaccharide lyases. **(A)** Heat map showing distribution of the CAZYmes distribution. Values are ln(*x* + 1)-transformed and rows and columns clustered using Euclidean distance and Ward linkage. **(B)** Principal components analysis showing the separation of the studied strains based on the distribution of peptidases and peptidase inhibitors. The ellipses are predicted to indicate probability (0.95) that a new observation from the same group will fall inside the ellipse.

The thirty-five Tremellales and the outgroup strains harbor 6,918 carbohydrate-active enzymes (CAZYmes) at an average of 192 proteins per proteome (Supplementary Table S2). The two soil isolates harbor the highest number of CAZYmes with 301 and 333 proteins in *S. podzolica* DSM 27192 and *Cryptococcus* sp. JCM 24511, respectively, while the genomes of the two dead arthropods associated *C. depauperatus* strains, CBS 7841T and CBS 7855 code for the least number of CAZYmes of 121 and 125 proteins, respectively. Consequently, the soil isolates alongside majority of the plant associated saprobes cluster separately from the rest of the strains based on the CAZYmes distribution (Figures 4A,B). Furthermore, the mycoparasitic and pathogenic isolates appear to share similar CAZYmes distribution relative to the sea and the arthropods frass isolates (Figures 4A,B).

Like the proteolytic enzymes, correlation analyses revealed significant (*P* < 0.01; *r* = 0.54) association between CAZYmes and proteome size and lack of association with genome size (Figure 3 and Supplementary Figure S2). Similarly, there was highly significant (*P* < 0.01) and strong association (*r* = 0.90) between CAZYmes abundance and predicted sugar transporters STs (Figure 3). The proteomes of the soil isolates, which harbor the highest number of CAZYmes, also contain more STs, comprising 275 and 284 proteins in DSM 27192 and JCM 24511, respectively. By contrast the rest of the strains harbor between 20 and 220 proteins with the proteomes of the dead arthropod associated strains showing the least STs of 20–22 proteins (Supplementary Figure S2).

##### Auxiliary activities (AA)

Evaluation of the CAZYmes datasets revealed 425 putative enzymes linked to ten auxiliary activities (AA) family (Supplementary Table S2). The soil isolates harbor relatively higher numbers of AA ranging between19 and 20 proteins and distributed in seven AA classes. Although more diverse with 8 AA classes, the pathogenic strains follow closely with 15–18 AA proteins. However, the distribution of AA among the plant saprobes ranged between 8 and 14 proteins indicating potential functional diversity among the various saprobes. The genomes of all the studied Tremellales code for orthologs of AA families AA1 (laccase-like multicopper oxidases), AA3 (glucose-methanol-choline (GMC) oxidoreductases family) and AA5_1 (glyoxal oxidase). The genomes of DSM 27192 and Phaff54-35 uniquely code AA2 (class II lignin-modifying peroxidases) in single copies while orthologs of AA9 (formerly GH61; copper-dependent lytic polysaccharide monooxygenases) are present in single copies uniquely among the three pathogenic alongside the two dead arthropods and one mycoparasitic (CBS 7805) strains.

##### Carbohydrate-binding modules (CBMs)

The genomes of the studied strains code for 341 proteins that contain CBMs with three, namely, CBM13 (associated with xylanase in fungi), CBM43 (usually associated with GH17 or GH72) and CBM48 (usually appended to GH13 or β subunit of AMPK) occurring in all strains (Supplementary Table S2). One protein each from JCM 2954^*T*^, NRRL Y-17943 and Phaff54-35 contain CBM20 (associated with glucoamylase in fungi), CBM35 (usually associated with xylanases) and CBM66 (associated with β-fructosidase reported in *Bacillus subtilis*), respectively. However, CBM67 (linked to α-L-rhamnosidase) is the most abundant module observed in the strains occurring nearly in 2-folds (12–14 proteins) among the two soil strains compared to a range of 0–7 proteins among the thirty-three other strains. However, of the 341 CBMs, 223 (65.4%) occur in CAZYmes, predominantly (115 CBMs) CBM67 in proteins with GH78 signatures while 118 CBMs occur in proteins without signatures of hydrolysis activity (Supplementary Table S3).

##### Carbohydrate esterases (CEs)

Genomes of the strains included in this study code for seven CEs with only CE4 being common to all strains (Supplementary Table S2). CE10 (arylesterase and others) was identified only in strain JCM 2954^*T*^, CE15 (4-O-methyl-glucuronoyl methylesterase) occur in two strains; JCM 24511 and Phaff54-35 while CE8 (pectin methylesterase) is unique to the two soil isolates DSM 27192 and JCM 24511, 05/00 (sea) and Phaff89-39 (unknown origin). The three *K. mangroviensis* (isolated from sea) and two saprobic isolates uniquely contain CE1 (wide range of esterases) while CE5 (acetyl xylan esterase and cutinases) occurs uniquely among five saprobic strains and one sea isolate. Furthermore, the sea isolates alongside two isolates each of arthropods frass, dead arthropods and plants lack orthologs of CE9 (*N*-acetylglucosamine 6-phosphate deacetylase).

##### Glycoside hydrolases (GHs)

Putative GHs proteins identified in the studied proteomes include 3,567 proteins distributed in 57 families, thus constituting the most abundant (∼51.2%) and diverse putative CAZYmes of the studied strains (Supplementary Table S2). Of the 57 GH families, 13 (GH105, GH13, GH133, GH16, GH17, GH18, GH3, GH37, GH47, GH5, GH71, GH72, and GH9) occur in at least one copy in all strains while 11 others (GH11, GH125, GH130, GH141, GH151, GH30_7, GH33, GH39, GH49, GH76, and GH97) show restricted distribution occurring only in between one to four strains. Consistent with overall CAZYmes profile, the proteomes of the two soil, strains DSM 27192 and JCM 24511 contain the highest number of 175 and 198 GHs, respectively, represented each in 45 families. However, relative to the rest of the studied strains, two plant associated isolates NRRL Y-17943 (143 GHs) and Phaff54-35 (160 GHs) show greater GHs diversity with 52 and 49 families, respectively. Despite the difference between the four strains above, they uniquely harbor GH125 (exo-α-1,6-mannosidase), GH30_7 (endo-β-1,4-xylanase and several other enzymes), and GH67 (α-glucuronidase or xylan α-1,2-glucuronidase) while the latter strains uniquely contain single copies of GH55 (exo-β-1,3-glucanase or endo-β-1,3-glucanase) and GH76 (α-1,6-mannanase or α-glucosidase). By contrast the soil associated isolates show overrepresentation of GH1 (β-glucosidase and several other enzymes), GH106 (α-L-rhamnosidase or rhamnogalacturonan α-L-rhamnohydrolase), GH3 (β-glucosidase and several other enzymes) GH43 (β-xylosidase and several other enzymes), GH5 (endo-β-1,4-glucanase/cellulase and several other enzymes) and GH78 (α-L-rhamnosidase, rhamnogalacturonan α-L-rhamnohydrolase and L-Rhap-α-1,3-D-Apif -specific α-1,3-L-rhamnosidase) by at least 2-folds relative to the rest of the strains.

##### Glycosyl transferase (GTs)

A survey of the predicted CAZYmes of the studied strains revealed 2,198 GTs grouped in 30 distinct families (Supplementary Table S2). Twenty-two GTs are represented in all studied proteomes with GT20, GT21, GT24, GT33, GT39, GT48, GT66 showing similar distributions. The soil isolate, JCM 24511 harbors the highest number of GTs of 76 distributed in 26 families but in terms of diversity, CBS 7118T, 05/00 and DSM 27192 each harbor 27 distinct GT families. GT2 (cellulose synthase, chitin synthase and numerous others) and GT90 (UDP-Xyl: (mannosyl) glucuronoxylomannan/galactoxylomannan β-1,2-xylosyltransferase, UDP-Glc: protein O-β-glucosyltransferase and UDP-Xyl: protein O-β-xylosyltransferase) with 434 and 269 proteins, respectively constitute 31% of the identified GTs while GT17 (β-1,4-mannosyl-glycoprotein or β-1,4-N-acetylglucosaminyltransferase) and GT49 (β-1,3-N-acetylglucosaminyltransferase) constitute the rarest GTs occurring only in two strains each. Notably GT57 (Dol-P-Glc: α-1,3-glucosyltransferase) and GT59 (Dol-P-Glc: Glc2Man9GlcNAc2-PP-Dol α-1,2-glucosyltransferase) are shared by eight strains including two soil isolates while the pathogenic strains along with four arthropods frass, two plants and one sea isolates harbor GT76 (Dol-P-Man: α-1,6-mannosyltransferase), which is missing in the latter group. This group, however, harbors GT57 and GT59. Alpha-1,6-mannosyltransferase, involved in cell wall α-1,6-mannose backbone extension, has been implicated in pathogenicity of *Candida albicans* (Zhang et al., 2016) and may play similar role among the studied pathogenic strains.

##### Polysaccharide lyases (PLs)

**FIGURE 5:**
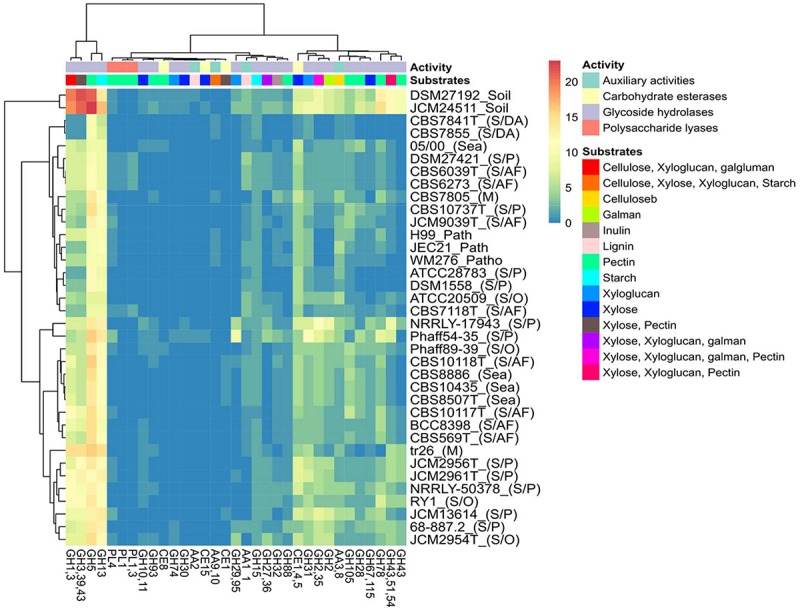
Heat map of the distribution of predicted polysaccharide hydrolyzing CAZYmes, activities and potential substrates among the Tremellales species. The various niches of the studied strains are enclosed parenthesis: S/AF, S/DA, S/P, M, S/O and patho representing saprobic (arthropods frass), saprobic (dead arthropods), saprobic (plants), mycoparasitic, saprobic (others) and pathogenic isolates, respectively.

Analysis of the putative CAZYmes of the studied Tremellales reveals 177 PLs belonging to seven families including PL0 (non-classified) present in all strains (Supplementary Table S2). Surprisingly, two families PL1_4 (exo-pectate or pectate lyase) and PL3_2 (pectate lyase) are exclusively present in four arthropods frass isolates. With exception of CBS 10118T and CBS 10117T, all the arthropods frass, dead arthropods and the pathogenic isolates lack PL22_2 (oligogalacturonate lyase/oligogalacturonide lyase). The pathogenic, dead arthropods and the sea isolates lack PL8_4 (hyaluronate lyase and others) and the latter two groups are missing the orthologs of PL4 (rhamnogalacturonan endolyase). The implication of various combinations of PLs and other CAZYmes (Figure 5) predicted to partake in complex biomass degradation is discussed below.

### Utilization of Carbon Substrates

**FIGURE 6:**
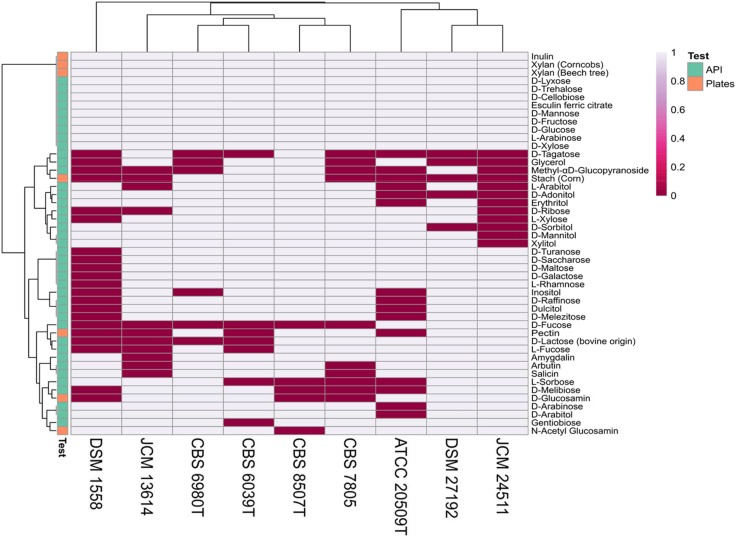
Carbon source utilization among nine Tremellales strains on API 50 strips and agar plates.

To assess the ability to assimilate various carbon sources and to deconstruct complex substrates, nine strains selected from distinct clades in the presented phylogeny (Figure 1), representing various habitat preferences were tested on API 50 CH strips and grown on agar plates containing eight polysaccharides and two monosaccharides. Results on 47 substrates for which at least one strain tested positive is summarized in Figure 6. All tested strains showed activity on eleven API substrates; D-cellobiose, D-fructose, D-glucose, D-lyxose, D-mannose, D-trehalose, D-xylose, esculin ferric citrate, inulin, and L-arabinose and grew on two of the eight polysaccharides; xylan (beech tree) and xylan (corncobs). The *K. mangroviensis* CBS 8507^*T*^ and *S. podzolica* DSM 27192 isolated from sea and soil, respectively, each grew on 42 substrates while *T. mesenterica* DSM 1558 (plant isolate) metabolized only 26 substrates. *S. podzolica* DSM 27192 utilized seven carbon sources (methyl-αD-glucopyranoside, D-ribose, L-arabitol, erythritol, L-xylose, D-mannitol, and xylitol) on which no activity was observed for its closest relative *Cryptococcus* sp. JCM 24511. Utilization of these seven substrates by DSM 27192 and the lack thereof by JCM 24511 further supports the evolutionary divergence of the two strains as predicted via phylogenomic analyses. Aside these, the two soil isolates utilize more substrates in common and uniquely use D-Fucose compared to the rest of the strains tested (Figure 6). The studied strains grew on five of the eight polysaccharides included in this study and no visible growth was observed on cellulose, CMC, and chitin (crab shell). Five strains, including the two soil isolates showed growth on pectin and only three strains *K. mangroviensis* CBS 8507T, *D. aurantiaca* CBS 6980^*T*^ (JCM 2956^*T*^) and *C. amylolentus* CBS 6039^*T*^ isolated from sea, plant, arthropods frass, respectively, grew on plates containing starch (corn).

## Discussion

This study provides an in-depth evaluation of the genetic relationship, diversification and biomass biodegrading potential of the Tremellales. We previously described the genome sequence of the oleagenic strain DSM 27192 (Aliyu et al., 2019) and in the current extended study, thirty-four other Tremellales have been incorporated with a view to further explore their genetic potentials. These isolates originated from a variety of habitats, potentially representing diverse niche specializations which could be explored for the identification of novel enzymes with wide ranges of biotechnological applications. Aside the diversity of isolation sources, the taxa included in this study (Table 1) are evolutionary diverse and based on a combination of genomics metrics the data provides additional insight into the application of phylogenomics for the delineation of these taxa.

### Phylogenomics Provides Insight Into the Evolutionary Relationships of the Tremellales

The presented phylogeny generated from 1,597 single copy orthologous proteins (Figure 1) largely agrees with the delineation of the Tremellales within the *Tremellomycetes* phylogenomic framework reported by Liu et al. (2015). Using taxa of known taxonomic standing, inferences were derived on the interrelationships between closely related strains. For instance, based on phylogeny and percent “nucleotide” identity values, Passer et al. (2019) proposed that CBS 6039^*T*^ and CBS 6273 are conspecific (*C. amylolentus*) while *C. wingfieldii* CBS 7118^*T*^ and *C. floricola* DSM 27421 represent distinct species. Similarly, previous phylogenetic analysis of the *C. gattii*/*neoformans* species complex has proposed seven species with the three strains included in this study belonging to three distinct species (Hagen et al., 2015). The AAI value of 88.8% shared between the two closely related soil and oleagenic isolates (Schulze et al., 2014; Tanimura et al., 2014) DSM 27192 and JCM 24511, affiliated with *S. podzolica* based on D1/D2 region of LSU rRNA gene compares well with the interspecific AAI range of *C. gattii* WM276, *C. neoformans* var. *grubii* H99 and *neoformans* JEC21 (86.3–90.0%), as well as that of *C. amylolentus* CBS 6039^*T*^, *C. wingfieldii* CBS 7118^*T*^ and *C. floricola* DSM 27421 (91.6–92.7%). This value is also lower than the intraspecific AAI of *C. amylolentus* species, CBS 6039^*T*^ and CBS 6273 (98.7%), *C. depauperatus* strains, CBS 7841^*T*^, CBS 7855 (96.2%) and *Kwoniella heveanensis* CBS 569^*T*^ and BCC8398 (96.7%) and the three *K. mangrovensis* strains, CBS 8507^*T*^, CBS 10435 and CBS 8886 (97.6%). Consequently, DSM 27192 and JCM 24511 may represent two distinct species. In the same vain, ATCC 28783 and DSM 1558 affiliated to *Tremella mesenterica* (AAI: 92.7%) apparently constitute two distinct species. However, the *T. mesenterica* strains share higher average nucleotide identity (ANI) of 96.73% compared to 86.52% for the *Saitozyma podzolica* strains (Supplementary Figure S1B). The latter strains also show greater genetic dissimilarity relative to *Cryptococcus neoformans* complex (ANI: 88.31%) or *C*. *amylolentus* and *C*. *wingfieldii* (ANI: 92.26), further supporting the possibility that they represent two distinct species. In addition to phylogeny, AAI and ANI values, these strains differ in genome sizes, predicted proteomes and numbers of strain specific proteins. However, the absence of the genomes of the type strains of these taxa may exclude any further speculations regarding genome-based taxonomy for the strains. While genomic metrics like AAI (Konstantinidis and Tiedje, 2005b), ANI (Konstantinidis and Tiedje, 2005a; Jain et al., 2018) and *in silico* DNA–DNA (Meier-Kolthoff et al., 2013, 2014) show remarkable power in delineating microorganisms and correlate well with the conventional lab-based DNA–DNA hybridization, often used in fungal taxonomy (Lachance, 2018), their application is yet to gain full traction in fungal taxonomy partly due to lack of baseline data that validate DNA–DNA reassociation used in fungal taxonomy (Takashima and Sugita, 2019).

### Tremellales Harbor Abundant and Diverse Carbohydrate-Active and Proteolytic Enzymes

To leverage functional capabilities associated with the diversity and niche specializations of the Tremellales for the identification of enzymes of potential industrial and biotechnological applications, proteomes of the studied strains were scanned for carbohydrate-active and proteolytic enzymes using MEROPS and dbCAN database, respectively. Several studies have highlighted the potential applications of carbohydrate-active and proteolytic enzymes and in some instances, the two groups of enzymes have been reported to function complementarily, for example, in the biodegradation of plant and enhanced plant fiber digestibility in the diet of ruminant (Cabaleiro et al., 2002; da Silva, 2017).

### Metallopeptidases and Cysteine Proteases Are the Most Abundant and Diverse Proteolytic Enzymes of Tremellales

The present data showed a strong correlation between peptidases and amino acid transporters both of which have been implicated in the regulation and metabolism of nitrogen compounds (Uemura et al., 2004; Abdel-Sater et al., 2011; Kohl et al., 2012; Shah et al., 2013). As previously suggested about nitrogen portioning for grain filling (Kohl et al., 2012), elucidation of the functional roles of both the transporters and specific peptidases in fungi could have practical implication toward further development of nitrogen limited function like oil accumulation.

Inhibitors of peptidases are of interest because of their potential applications in various fields, including but not limited to medicine, crop protection and biotechnology (Sah et al., 2006; Sabotiè and Kos, 2012). They are widely distributed protein families that control the activity of peptidases. However, inhibitors of cysteine peptidases show restricted distribution in fungi (Sabotiè and Kos, 2012). The caspase (C14) inhibitor I32 belongs to a group of inhibitor of apoptosis proteins (IAP) which regulate a wide range of cellular activities (Dubin, 2005; Sah et al., 2006; Dunaevsky et al., 2014). Although, the antiapoptotic activity of I32 homologs has been demonstrated in *S. cerevisiae*, the inhibitory effects on C14 has not been proven (Walter et al., 2006; Owsianowski et al., 2008). The presence of C14 in the proteomes of all studied strains and restriction of I32 to four (mainly free living) strains may support the lack of association of the two proteins in fungi but instead, suggest a probable role in specific survival strategy in dynamic environments such as soil. Similarly, the precise function of the serine carboxypeptidase Y inhibitor (I51) remains largely obscure (Dunaevsky et al., 2014) but linked to various environmental stress responses such as heat shock and oxidative stress (Sabotiè and Kos, 2017). Unlike I32 and although, the homologs of the putative target S10 (serine carboxypeptidase) are prevalent in all the studied genomes, only 16 genomes, including those of the three sea (*K. mangroviensis* CBS 10435, CBS 8507^*T*^ and CBS 8886) and one plant (*N. encephala* UCDFST 68-887.2) isolates, harbor the specific serine carboxypeptidase Y (S10.001: MER0002010) homologs inhibited by I51. Thus, I51 may play multiple roles including responses to environmental stress and specifically nutrient limitation (Parzych et al., 2018) among the strains harboring serine carboxypeptidase Y.

Among the predicted peptidases, two families, glutamic (G) and mixed (P) peptidases show restricted distribution with the former encoded on the genome of one soil isolate and the latter present in only four plant related isolates. Glutamic peptidases are among the well-studied fungal proteolytic enzymes to be identified and show restricted distribution among fungal taxa (da Silva, 2017). Specifically, the aspergilloglutamic peptidase (G01) first discovered in *A. niger* var. *macrosporus* and named eqolisins (Kataoka et al., 2005) has found various industrial applications, including a role as hydrolyzing agent against various inhibitors present in numerous components of animal feed (Bruins et al., 2018) and dietary supplement (Bruins et al., 2019). Thus, the prediction of a putative G01 protein in DSM 27192 highlights the biotechnological potential of this strain that requires further investigation. P1 or β-aminopeptidases include aminopeptidases and self-processing peptides that are of biotechnological and pharmaceutical interest for cleavage of β− and mixed α,β−peptides and amides. Orthologs of P1 has been identified in a wide range of prokaryotes and eukaryotes, including fungi (Heck et al., 2006; Hiraishi, 2016; John-White et al., 2017). However, the natural substrates for these peptidases are not known and therefore their restriction to only a few plant isolates could be associated with their potential capacity to use synthetic β-peptidase (John-White et al., 2019) and hence characterizing these proteins will expand the limited knowledge regarding their function.

Non-animal aspartic peptidases sources such as fungi are of special biotechnological interest as cheap alternative to chymosin obtained from the stomach of calves (Kumar et al., 2010; da Silva, 2017). They are mainly applied in food industry, for instance, in cheese processing (Kumar et al., 2010; Yegin et al., 2011). Prevalence of the A01A family, which is secreted aspartic proteases, among the studied isolates, especially those from arthropods frass may underpin the role of these enzymes in proteolytic activities associated with both general nutrients acquisition and perhaps pathogenesis among the pathogenic strains (Monod et al., 2002; Mandujano-Gonzalez et al., 2016).

Scanty information is available about the functional role of C-peptidases in fungi (da Silva, 2017). However, these enzymes have been associated with parasitism in several organisms, including fungi and hence considered to be of clinical importance (Barrett and Rawlings, 1996; Atkinson et al., 2009). For example, expression of C14 (caspase) has been reported in parasitic microorganisms including fungi (Atkinson et al., 2009; McLuskey and Mottram, 2015). However, the genomic data presented here showed similar distribution of these enzymes among all the studied strains.

M-peptidases such as matrix metallopeptidases (MMPs) have potential applications in cancer and arthritis therapy because of their ability to degrade extracellular matrix (Rao et al., 1998). However, orthologs of MMPs have been reported to occur rarely in fungi, strictly phylum Ascomycota and no activity data are available (Cerdà-Costa and Gomis-Rüth, 2014; Marino-Puertas et al., 2017). M-peptidases have also been implicated as virulence factors in various organisms (Gravi et al., 2012). However, this study could not establish enrichment of any of these peptidases in either the pathogenic or the parasitic relative to the saprobic isolates.

Although shown to be among the most prevalent peptidases (da Silva, 2017), this study revealed that serine peptidases are less abundant and less diverse compared to the metallopeptidases and cysteine proteases. Consistent with previous studies (Muszewska et al., 2017), serine peptidase abundance appears to be significantly associated with proteome size (Supplementary Figures S5A,B). However, contrary to previous reports where plant associated taxa have been shown to harbor more orthologs of S9, S10, S12, S33, and S53 (Muszewska et al., 2017), this study revealed predominance of orthologs of S9 among the isolates obtained from soil (JCM 24511 and DSM 27192), rotten beech (JCM 13614) and a fungus (tr26) relative to isolates from plants and insect frass (Supplementary Figure S3C). Furthermore, in contrast to previous reports (Muszewska et al., 2017; Palmer et al., 2018) the distribution of S8 (subtilisin) does not appear to be shaped by niche specialization among the studied Tremellales.

Previous reports have indicated that T-peptidases are more abundant among aquatic and soil inhabiting Bacteroidetes (Nguyen et al., 2019). The fungi studied here, however, appear to show similar distribution of T-peptidases.

### Carbohydrate-Active Enzymes Enzyme (CAZyme) Repertoire and Substrate Utilization Among Thirty-Five Tremellales

Evaluation of the predicted proteomes of the thirty-five Tremellales isolated from different environments revealed greater CAZYmes abundance among the soil isolates similar to numbers reported from previous studies of representative fungi, where more than 50% of the strains harbor >300 CAZYmes (Zhao et al., 2014). However, CAZYmes distribution among the majority (thirty-three) of the studied strains falls within the lower 50% range and as observed previously, modes of life do not appear to be associated with abundance or diversity of CAZYmes (Zhao et al., 2014). Further reflecting their versatility, the soil isolates also harbor greater number of sugar transporters (STs). STs distribution have been correlated with fungal capability to utilize carbon sources with greater numbers of STs typically observed among metabolically versatile fungi capable of using broader range of substrates (Cornell et al., 2007). In addition to sugar transduction, certain STs also regulate the expression of CAZYmes such as cellulase and xylanases (Zhang et al., 2013; Huang et al., 2015) and have therefore been considered veritable targets for strain improvement in various biotechnological applications (Borin et al., 2017).

Although CBMs are most abundant among the soil isolates, majority of the proteins containing these modules do not have CAZYmes modules. CAZYmes without CBMs have been reported to show poor catalytic activities relative to their CBMs associated counterparts (Pasari et al., 2017). To enhance catalytic activity of certain CAZYmes lacking CBMs, the latter modules could be engineered in the enzymes. For instance, improved hydrolytic activities have been detected with CBM augmented *M. albomyces* cellulases (Szijártó et al., 2008). Non-hydrolytic proteins containing CBMs, however, enhance substrate degradation by participating in a multienzyme complex called cellulosome whose catalytic efficiency is greatly reduced without these proteins (Shoseyov et al., 2006). CBMs are important targets of enzyme engineering for enhanced hydrolytic activities via the construction of hybrid enzymes (Kim et al., 1998; Limón et al., 2001; Shoseyov et al., 2006) or hydrolytic scaffolds such as the cellulosome. Detection of CBMs in proteins with no known CAZYme modules could potentially signify unknown hydrolytic proteins.

The two *Saitozyma* isolates from soil also harbor more proteins linked to auxiliary activities, probably indicating a greater lignin-degrading capacity (Whittaker et al., 1996). However, some of the specific AA families such as AA2 which is associated with lignin modification (Lundell et al., 2010), occur uniquely, albeit in single copies, in two of the studied strains, signifying their putative specialty in plant biomass degradation. In contrast, AA9 associated with cellulose and hemicellulose utilization (Hemsworth et al., 2013; Riley et al., 2014), has been identified across pathogenic, symbiotic and saprobic fungi. However, involvement of AA9 in host invasion has been suggested in *Pyrenochaeta lycopersici* (Valente et al., 2011) and hence a link to this trait among these strains could be speculated. Similarly, AA8 (iron reductases) is exclusively present in the pathogenic, soil and four saprobic isolates. The pathogenic isolates also have two copies of AA6 (1,4-benzoquinone reductase) along with one arthropods frass strain while six other saprobic strains have only single copies of AA6. AA8 is suggested to be involved in the production of reactive hydroxyl radical potentially associated with non-enzymatic breakages of cellulose and hence a component of plant biomass degradation proteins while AA6 is involved in degradation of aromatic compounds intracellularly and serve as protection against reactive quinone compounds in fungi (Levasseur et al., 2013).

Aside CE4, the largest family of CEs for which known activities include acetyl xylan esterases, chitin deacetylase, peptidoglycan GlcNAc deacetylase, among others (Nakamura et al., 2017), that is shared by all the studied strains, the studied proteomes showed variation in terms of CEs distribution and potential biotechnological potentials. For instance, the recently discovered CE15 occurs in only two strains and has the unique ability to cleave the ester bond between lignin and glucuronic acid residues of glucuronoxylans (Arnling Bååth et al., 2016; Mosbech et al., 2018). However, most of the CEs identified in this study have been reported to show wide range of substrate specificity. For example, most CE10 CAZYmes do not act on carbohydrates substrates (Zhao et al., 2014). Despite this, the diversity of the CEs represents enormous potential in various fields, for instance, CE1, present in four strains, includes enzymes of potential biomedical application as drug design targets (Nakamura et al., 2017).

Because of their important role in breaking down plant cell walls, GHs are among the most characterized enzymes of fungal origin (Murphy et al., 2011). Majority of GHs shared by all the studied Tremellales are among those reported to be widely distributed in fungi and the overrepresentation of GH2, GH13, and GH16 is also consistent with reports on the top most abundant fungal GHs (Zhao et al., 2014). The current study showed varied distribution and abundance of GH among the thirty-five studied Tremellales and different life styles with the two soil isolates harboring greater numbers but less diverse GHs relative to two plant associated strains, thus indicating that the latter group may have greater substrate range. Interestingly, GH30, GH67, and GH125 shared by the four (soil and plant) isolates occur rarely among previously studied *Basidiomycota* with GH67 only found among the Ascomycetes (Zhao et al., 2014).

Despite prediction of CAZYmes repertoire that could potentially act on numerous substrates (Figure 5), the nine tested isolates could not utilize cellulose (carboxymethylcellulose; CMC) and chitin. GH18, GH19, and GH85 and AA10 are chitinases involved in the degradation of chitin (Lombard et al., 2014; Berlemont, 2017). The inability of these strains to utilized chitin may be explained by the absence of the complete set of chitinases as only GH18 orthologs are present in the proteome of all tested strains. Of the three cellulose active GHs sets (Coutinho et al., 2009; van den Brink and de Vries, 2011), all nine strains lacked β-1,4-endoglucanase (GH5,GH7,GH12 and GH45) and cellobiohydrolase (GH6 and GH7) but harbor the orthologs of β-1,4-glucosidase (GH1 and GH3). In addition, *S. intermedium* CBS 7805 harbors AA9, a module known for its cellulolytic activity (Langston et al., 2011). It is not surprizing, however, that these strains could not utilize this substrate since GH1, GH3, and AA9 alone may not be adequate for cellulose deconstruction as a combined action of several cellulases is essential for the complete breakdown of this polysaccharide (Singh et al., 2017). Several GH and AA proteins identified in plant biomass-degrading fungi, including AA9 have been reported to contain CBMs (Várnai et al., 2014; Berlemont, 2017). For instance, CBM1, which is cellulose targeting module has been reported occur at least once in ∼79% of 1,425 multi-domain CAZYmes (Berlemont, 2017). However, majority of AA9 proteins of several plant biomass-degrading fungi do not harbor CBM1 (Várnai et al., 2014). Regardless, the observed lack cellulolytic activity in all nine strains, including CBS 7805 may also be associated with the absence of these modules.

On the other hand, the proteomes of the nine strains contain numerous CAZYmes (Figure 5) associated with xylan decomposition (Coutinho et al., 2009; van den Brink and de Vries, 2011; Nagy et al., 2015), including acetyl xylan esterase (CE1,4,5), α-L-arabinofuranosidase (GH43,51,54) and β-1,4-D-xylosidase (GH3,39,43) that are present in all the isolates. By contrast, only three of the nine tested strains harbor β-1,4-D-Endoxylanase (GH10,11) (Coutinho et al., 2009; van den Brink and de Vries, 2011; Nagy et al., 2015). Despite these differences, all strains utilized the two xylan sources (beech tree and corncobs) as only carbon source. All strains were also able to grow on inulin. However, the current analysis did not predict inulinase (GH32) in two strains, DSM 1558 and JCM 13614.

Contrary to the above two scenarios, the proteomes of all tested strains (Figure 5) include orthologs of unsaturated rhamnogalacturonan hydrolase (GH105), β-1,4-D-galactosidase (GH2,35), β-1,4-D-xylosidase (GH3,39,43) and β-1,6-endogalactanase (GH5) and except DSM 1558 (no growth on pectin) orthologs of endopolygalacturonase or exopolygalacturonase or rhamnogalacturonan galaturonohydrolase or rhamnogalacturonan hydrolase (GH28), α-L-arabinofuranosidase (GH43,51,54) and α-rhamnosidase (GH78), all of which partake in the disintegration of pectin (van den Brink and de Vries, 2011; Nagy et al., 2015). Despite the range of pectinolytic enzymes, only five of the nine strains showed growth on pectin. Although, CEs; feruloyl esterase (CE1) and pectin methyl esterase (CE8) and PLs are known to participate in degradation of pectin components (van den Brink and de Vries, 2011), strains lacking one or both enzymes grew on pectin. Similarly, all tested strains harbour α-amylase (GH13), α-1,4-D-glucosidase/α-D-xylosidase (GH31) and glucoamylase (GH15), a set of CAZymes known to hydrolyze starch (van den Brink and de Vries, 2011; Nagy et al., 2015). However, majority of the strains sharing similar starch-degrading enzymes to those in isolates capable of hydrolyzing starch could not grow on it. The inability of strains harboring sets of polysaccharides hydrolyzing enzymes to breakdown the substrate may be associated with poor substrate binding capacity of the enzymes or low rate of enzyme activity which could also be shaped by numerous factors including growth conditions. Subsequent work will therefore focus on characterization of a selection of the predicted proteolytic and carbohydrate active enzymes from the soil inhabiting *Saitozyma* strains with emphasis on enzymes of potential novelty both in terms of structure and perhaps function.

## Conclusion

Considering pending economical, societal, and environmental challenges regarding the reduction of greenhouse gas emissions and the highly controversial “food-or-fuel” debate, it is highly important to enable competitive processing of renewable alternatives over fossil oil based or non-sustainable products. This can be achieved via identification and deployment of biomass deconstruction enzymes to generate the raw materials to produce biofuel and other industrial chemicals. This study aimed to provide an insight into the evolutionary relationships and functional diversification, with emphasis on biomass deconstruction capabilities, among thirty-five members of the order Tremellales. Contributing to the debate on genome-based circumscription of fungal isolates, our work revealed evolutionary distinction of two closely related, soil and oleagenic strains (*Saitozyma* species) both at genomic and phenotypic levels. We also identified 6,918 putative CAZYmes and 7,066 peptidases belonging to various families of the enzymes. The *Saitozyma* species, which have been isolated from soil harbor the largest numbers of both CAZYmes and peptidases. Although, the soil isolates show greater abundance of these feature, the limited number of soil isolates, and the lack of obvious trend among taxa of plant and arthropods origin, indicates that the distribution of these enzymes may not be associated with specific habitat type. Our future work includes isolation and characterization of the predicted biomass degrading enzymes from available fungal isolates using a range of analytics with a view to develop a robust enzyme cocktails with potential biotechnological applications.

## Data Availability Statement

Publicly available datasets were analyzed in this study. This data can be found in the NCBI or JGI databases: ASM18594v1, CNA3, ASM9104v1, ASM635230v1, ASM614915 v1, Cryp_amyl_CBS6039_V3, Cryp_amyl_CBS6273_V2, Filo_ depa_CBS7841_V1, Filo_depa_CBS7855_V2, Cryp_heve_BC C8398_V1, Cryp_heve_CBS569_V2, Cryp_deje_CBS10117_V1, Cryp_pinu_CBS10737_V1, Cryp_best_CBS10118_V1, Kwon_ mang_CBS10435_V2, Kwon_mang_CBS8507_V2, Kwon_ma ng_CBS8886_V1, ASM394221v1, JCM_24511_assembly_v001, JCM_2961_assembly_v001, JCM_2956_assembly_v001, Trem_ mese_ATCC28783_V1, Treme1, ASM98790v1, Phaff89-39v1.0, Treen1, Phaff54-35v1.0, Kocim1, JCM_2954_assembly_v001, CBS7805v1.0, Cf_30_300r_Split10plusN, ASM73882v1, JCM_90 39_assembly_v001, JCM_13614_assembly_v001, Mo29, ASM17 1244v1.

## Author Contributions

HA, OG, AN, and KO co-conceived the study and designed the experiments. HA, OG, XZ, and KO, performed the experiments and analyzed the data. HA, OG, and KO drafted the manuscript. All authors contributed to the final manuscript.

## Conflict of Interest

The authors declare that the research was conducted in the absence of any commercial or financial relationships that could be construed as a potential conflict of interest.

## Footnotes

1 https://github.com/dparks1134/CompareM
