# Supplementary material for: In silico Proteomic Analysis Provides Insights Into Phylogenomics and Plant Biomass Deconstruction Potentials of the Tremelalles
Source: Front Bioeng Biotechnol. 2020 Apr 3;8:226. doi: 10.3389/fbioe.2020.00226 (PMC7147457; doi:10.3389/fbioe.2020.00226)
Supplement: Supplementary file 4 [file Data_Sheet_1.PDF]

A

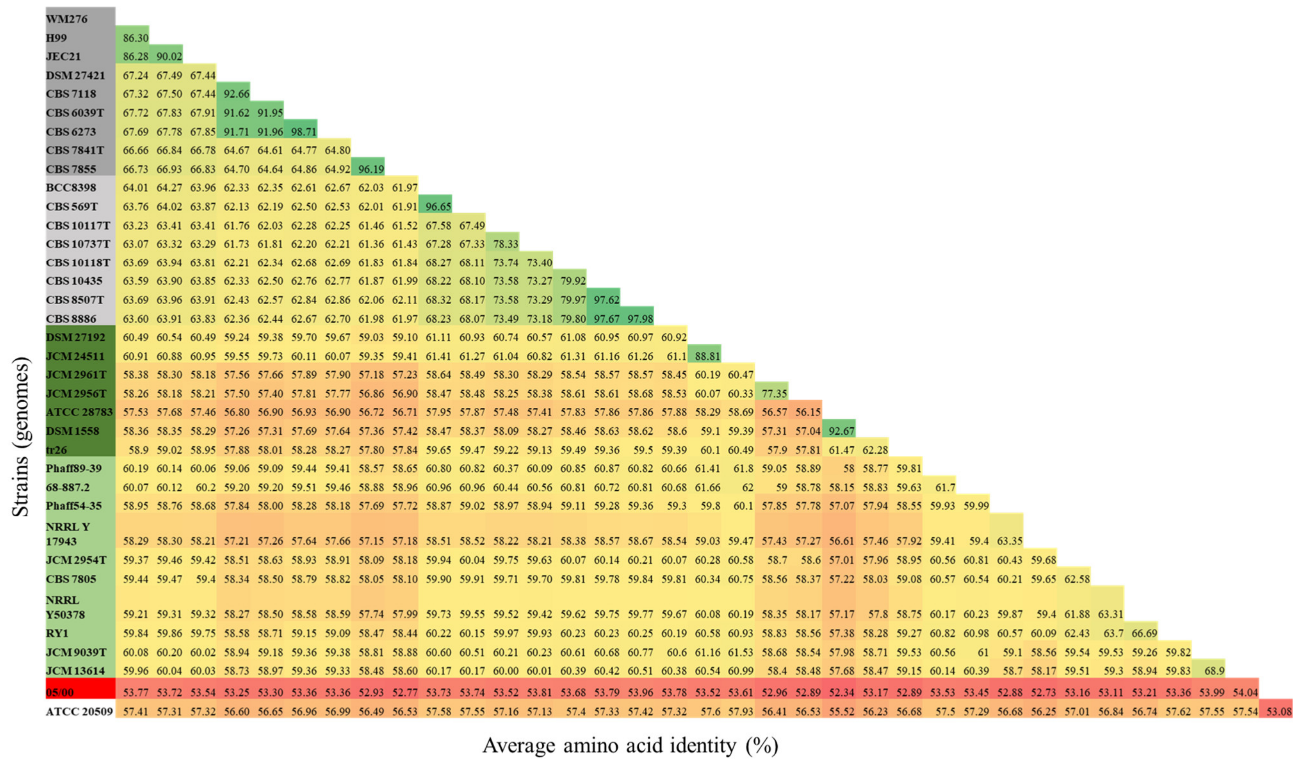

B

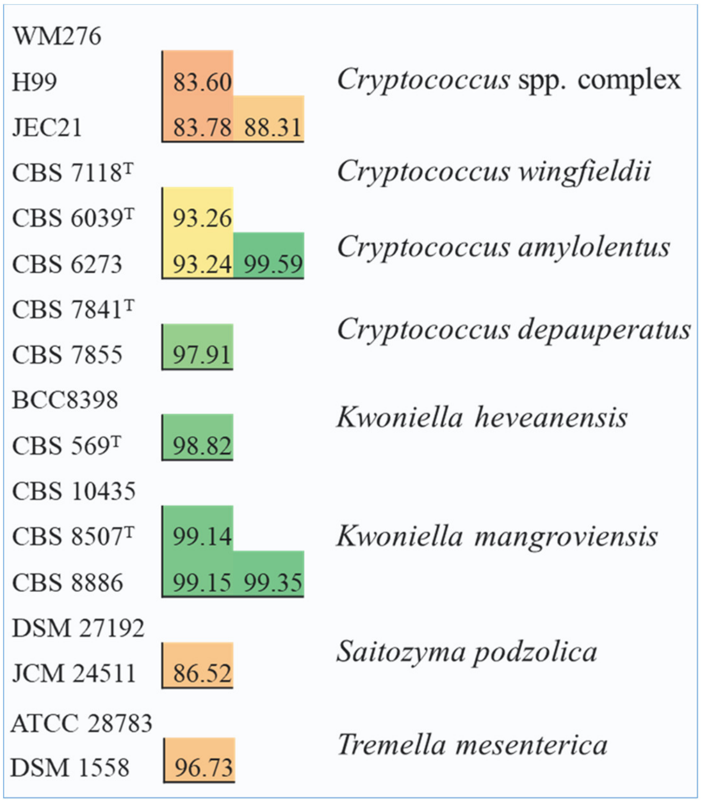

Figure S 1: A) Comparison of average amino acid identities (%) among thirty-five strains of Tremellales and one outgroup strain. The major clusters from the whole genome phylogeny have been colour coded. **B)** Comparison of average nucleotide identities (%) among selected members of the order Tremellales.

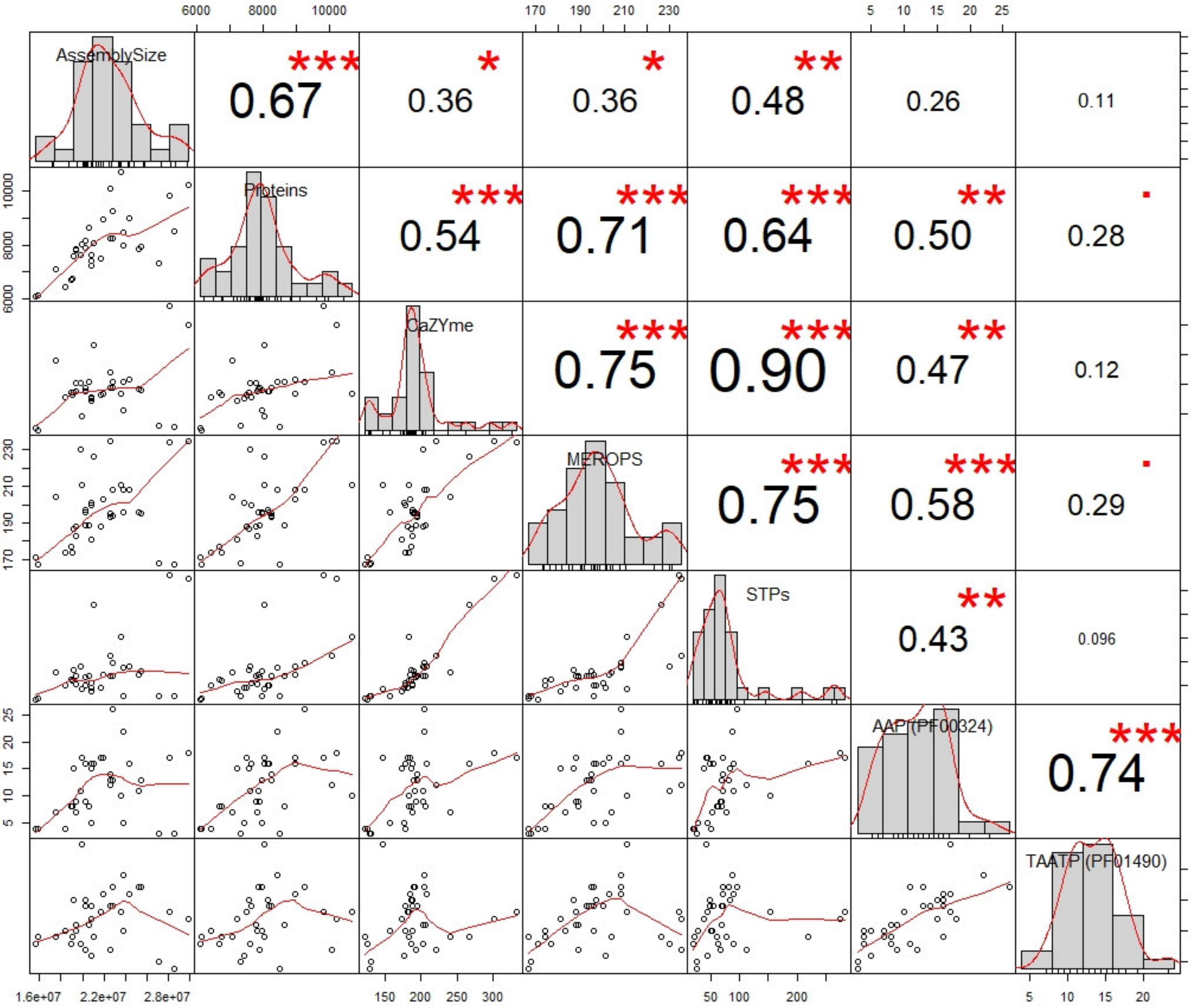

Figure S 2: Correlation analysis of genome and proteome sizes and predicted CAZYmes, peptidases, sugar, and amino acid transporters of the thirty-five Tremellales. *** indicate highly significant correlation (P > 0.01) with coefficient of correlation R values indicating the strength of the association.

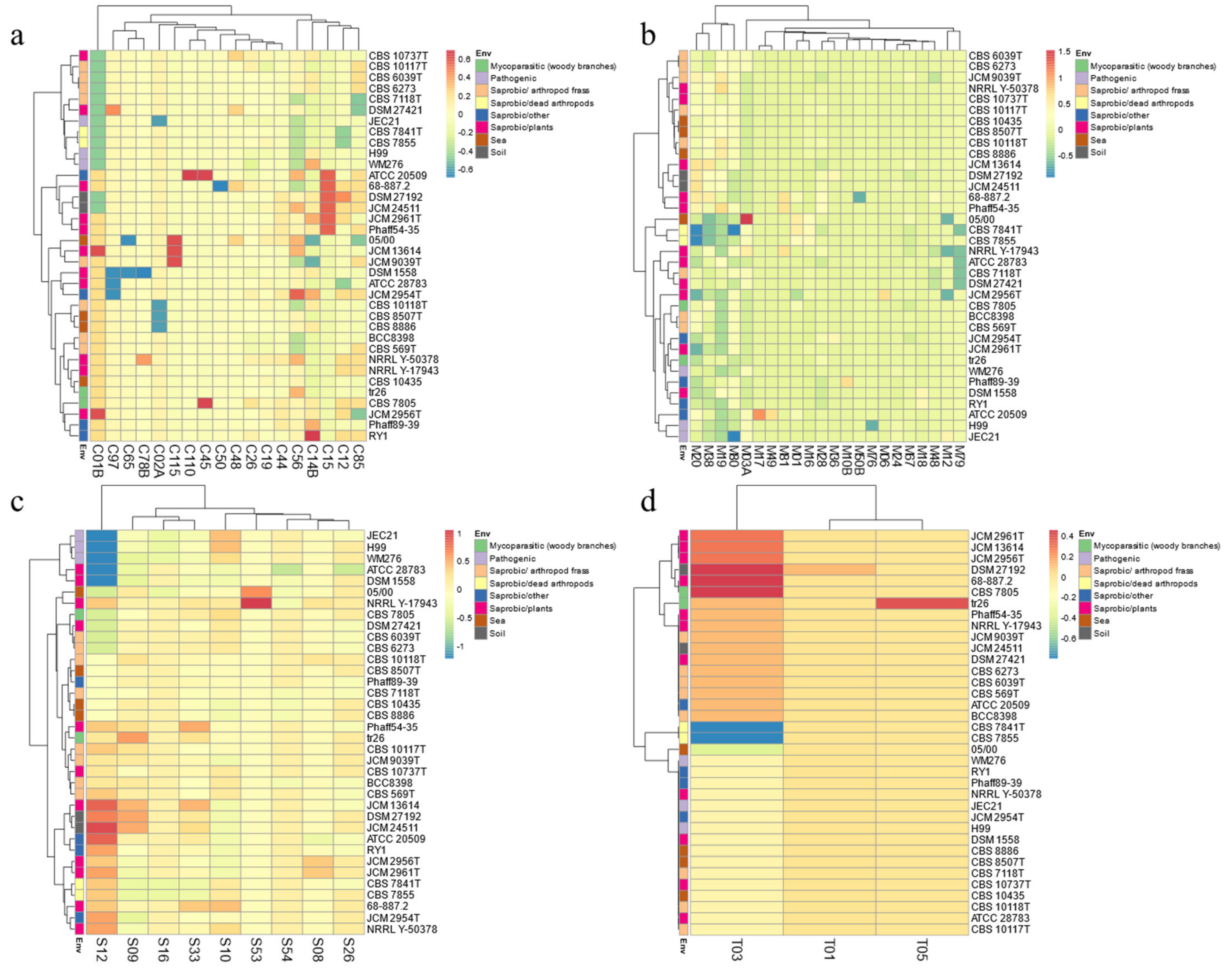

Figure S 3: Heat maps showing the distribution of peptidases families among the Tremellales species. **A)** cysteine peptidases, **B)** metallo-peptidases, **C)** serine peptidases and **D)** threonine peptidases. Values are ln(x + 1)-transformed and rows and columns clustered using Euclidean distance and Ward linkage.

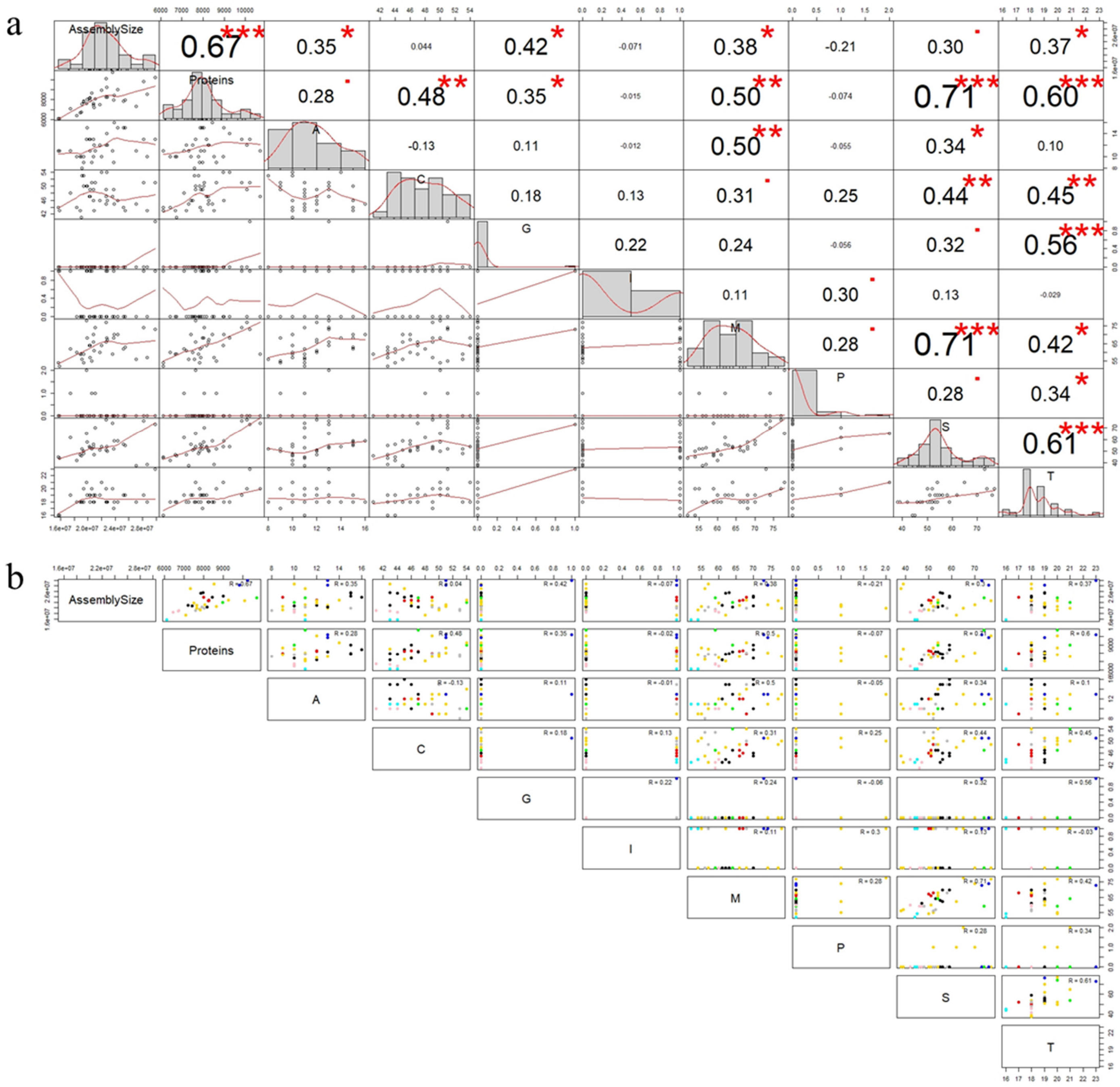

Figure S 4 Correlation analysis of genome and proteome sizes and predicted peptidases and peptidase inhibitor among the thirty-five Tremellales. **A)** *** indicate highly significant correlation (P > 0.01). **B)** Coefficient of correlation R values indicates the strength of the association and niche specialization of the strains are colour coded; black, blue, cyan, gold, green, grey, pink, red representing saprobic (arthropods frass), soil, saprobic (dead arthropods), saprobic (plants), mycoparasitic, saprobic (others), pathogenic, sea isolates, respectively.

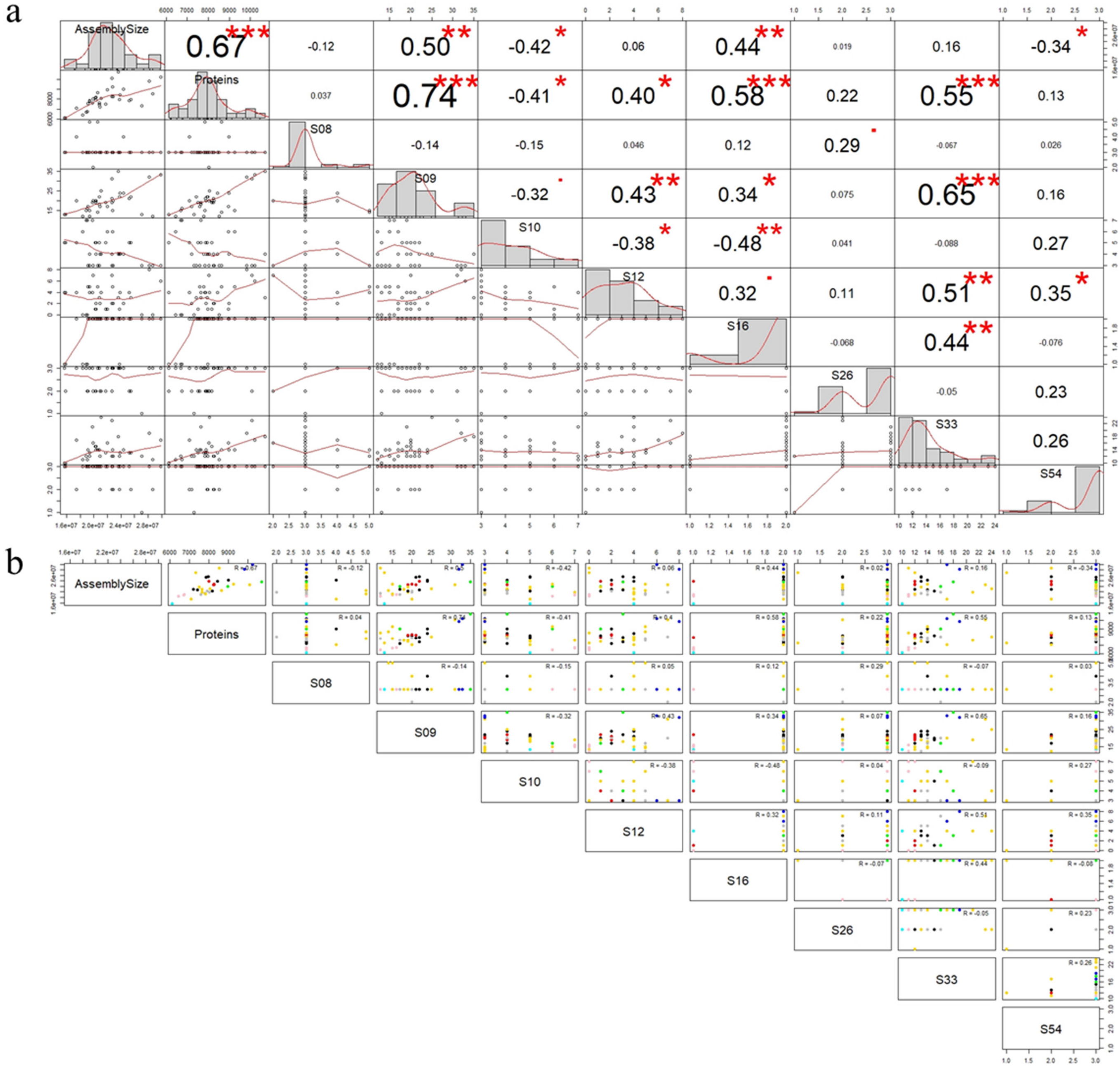

Figure S 5: Correlation analysis of genome and proteome sizes and predicted serine peptidase among thirty-five Tremellales. **A)** *** indicate highly significant correlation (P > 0.01). **B)** Coefficient of correlation R values indicates the strength of the association and niche specialization of the strains are colour coded; black, blue, cyan, gold, green, grey, pink, red representing saprobic (arthropods frass), soil, saprobic (dead arthropods), saprobic (plants), mycoparasitic, saprobic (others), pathogenic, sea isolates, respectively.
